# Decoding Cellular Complexity in the Gastric Precancerous Field: Spatial Evolution from Metaplastic Glands to Early Gastric Cancer

**DOI:** 10.3390/cells15212020

**Published:** 2026-11-05

**Authors:** Jingwei Kang, Shigang Ding, Xingting Guo

**Affiliations:** 1 Department of Gastroenterology, Peking University Third Hospital, Beijing 100191, China; kangjw2025@126.com; 2 Beijing Key Laboratory for Helicobacter Pylori Infection and Upper Gastrointestinal Diseases (BZ0317), Beijing 100191, China

**Keywords:** gastric precancerous lesion, gastric intestinal metaplasia, early gastric cancer, spatial heterogeneity, field cancerization, *Helicobacter pylori*, multicellular neighborhoods

## Abstract

**Highlights:**

**What are the main findings?**

- Gastric precancer is a spatially heterogeneous field composed of clonally distinct glands embedded within uneven microbial, immune, stromal and metabolic environments.
- Progression to early gastric cancer follows a branched and probabilistic process: metaplastic clones may expand and diversify, but only a minority may cross the malignant threshold, potentially influenced by local tissue conditions.

**What are the implications of the main findings?**

- Spatial proximity and transcriptional similarity alone cannot establish precursor–tumour ancestry; spatial profiling should be integrated with genomic lineage reconstruction.
- Mapping high-risk glands together with their multicellular neighborhoods may inform biopsy strategies, cancer-risk stratification and surveillance after endoscopic treatment, subject to prospective validation.

**Abstract:**

Gastric precancer is conventionally staged as a linear histological sequence, but it develops within a patchy, continuously remodeling mucosa. This narrative review reframes gastric carcinogenesis as a multiscale spatial process in which epithelial states, clonal gland dynamics, immune–stromal neighborhoods, regional *Helicobacter pylori* (*H. pylori*) and non-*H. pylori* colonization, and metabolic or physicochemical gradients may jointly shape local risk. Gland-resolved studies show that metaplasia may expand through fission and remain clonally continuous with dysplasia in selected lesions, whereas broad genomic and epigenetic field alterations may also support independent tumor emergence. Single-cell and spatial analyses identify hybrid, stem-like, and proliferative epithelial states embedded within evolving multicellular niches, although most evidence remains cross-sectional and biased toward established cancer. We therefore propose a non-deterministic model in which injury drives gland loss and niche remodeling; metaplastic clones expand and diversify; local ecological pressures may select high-risk states; and only a minority of glands cross the neoplastic threshold. This remains a working hypothesis, particularly for niche-driven selection of human precancerous clones. Clinically, the framework is consistent with the prognostic value of metaplastic extent, corpus involvement, and the Operative Link on Gastritis Assessment (OLGA) and Operative Link on Gastric Intestinal Metaplasia Assessment (OLGIM) systems, while defining the longitudinal, topographically annotated, gland-resolved studies needed to determine whether spatial risk maps add clinical value beyond established staging.

## 1. Introduction: From Precancerous Lesions to Spatially Organized Fields

Intestinal-type gastric carcinogenesis is commonly described as a multistep process progressing from chronic inflammation and glandular atrophy to intestinal metaplasia (IM), dysplasia, and invasive carcinoma [1]. This sequence remains clinically useful, but it is easily mistaken for a uniform and deterministic march across the gastric mucosa. In practice, the stomach contains sharply unequal risks within the same patient: normal-appearing glands can sit beside atrophic units, complete and incomplete IM, dysplasia, and occult neoplasia. Gastric cancer also remains a major global cause of cancer mortality, which makes the problem of identifying the small fraction of precursor-bearing patients who will progress especially consequential [2].

Prospective and population-based studies show that risk rises with the severity, extent, and topographic distribution of atrophy and IM [3,4,5,6]. Incomplete IM carries greater risk than complete IM, whereas corpus involvement and advanced Operative Link on Gastritis Assessment (OLGA) or Operative Link on Gastric Intestinal Metaplasia Assessment (OLGIM) stages identify a field that is both more extensive and biologically more advanced [3,5,6,7]. These observations already contain a spatial argument: a biopsy diagnosis is not merely a label attached to an isolated lesion, but a partial sample of a heterogeneous mucosal field. The updated European guidance therefore retains systematic topographic biopsy sampling and risk-adapted surveillance rather than treating a single negative biopsy as proof that the stomach is uniformly low risk [8].

Molecular work has strengthened this interpretation. Individual metaplastic glands can be clonal, clonally related glands can expand by gland fission, and genetically altered IM can occupy sizeable patches. However, clonal expansion alone does not imply that every altered gland is destined for malignant transformation [9,10,11,12,13]. Longitudinal genomic profiling further shows that high-risk IM may harbor persistent driver events, copy-number abnormalities, and distinct clonal or lineage trajectories. Complementary transcriptomic, histological, and microbiome studies further associate high-risk lesions with specific epithelial programs, inflammatory states, and microbial communities [11,12,14]. These findings motivate a spatial model, while leaving the relative contribution of clonal and environmental factors unresolved.

Single-cell and spatial technologies resolve epithelial states and tissue organization that bulk profiling averages across compartments [12,15,16,17,18,19,20,21,22]. Their inferential strengths differ: dissociation identifies cell states but loses native coordinates; spatial assays retain location but may mix neighboring cells; genomic lineage reconstruction tests ancestry but does not establish which local signal caused transformation. A cancer-adjacent metaplastic gland may be a related precursor, an independently altered lineage, or a bystander. Combining these methods is informative only when their distinct limitations are retained.

This review develops a gland-centred, multiscale framework for understanding the gastric precancerous field. After defining gland-level clonal evolution, it examines epithelial-state heterogeneity, multicellular microenvironments, *Helicobacter pylori* (*H. pylori*) gland colonization, non-*H. pylori* microbial geography, and metabolic and physicochemical pressures before considering the transition to early cancer. The central proposition is that malignant transformation may emerge when a subset of clonally altered glands encounter a permissive local ecology within a broader atrophic field. This formulation links classical pathology to spatial biology while maintaining the distinction between coexistence, common ancestry, and causal progression.

### Literature Scope and Evidence Interpretation

This narrative review was assembled from the topic-focused literature on gastric precancer, gland clonality, spatial profiling, host–microbial interactions, and clinical surveillance. Targeted PubMed/MEDLINE and publisher-site searches were supplemented by reference-list checking; key evidence was rechecked during revision on 19 October 2026. The cited literature spans 1988–2026. Searches combined gastric or stomach with intestinal metaplasia, precancer, gland clonality, spatial transcriptomics, cellular neighborhoods, microbiota, metabolism, and surveillance. Priority was given to primary human studies with anatomical or longitudinal information, relevant experimental perturbations, and clinical guidelines. Tumor-only studies were retained to identify hypotheses for precancer and are labeled accordingly. This was not a systematic review; no exhaustive retrieval, pooled estimate, or formal risk-of-bias score is claimed.

We distinguish anatomical localization, clonal ancestry, temporal association, and experimental perturbation. Higher spatial resolution does not automatically provide stronger causal evidence. Field cancerization denotes a mucosal territory containing altered gland populations with increased neoplastic susceptibility, not necessarily one clone. High-risk epithelial state denotes a candidate molecular phenotype associated with advanced histology or neoplasia unless prospective prediction is explicitly demonstrated. A spatial neighborhood is a prespecified anatomical or proximity-based grouping of cells; an ecotype inferred from dissociated cell abundance is not necessarily such a neighborhood. A permissive niche denotes a proposed local environment supporting persistence or progression. Deoxyribonucleic acid (DNA)-based ancestry, expression state, and future clinical risk are evaluated separately.

## 2. From Individual Glands to an Evolving Precancerous Field

### 2.1. What Gland Lineage Studies Establish

Isolated-gland analysis resolves molecular differences that pooled biopsies can obscure [9]. Mitochondrial lineage marking supports metaplastic gland clonality, fission-mediated patch expansion, and shared ancestry between metaplastic and dysplastic glands in selected lesions [10]. These studies provide direct anatomical and lineage information, but retrospective reconstruction samples surviving clones and cannot quantify the prospective cancer risk of every gland. A shared founder establishes relatedness, not which sampled gland gave rise to the tumor or which exposure drove that transition.

Copy-number profiling provides a complementary view. IM glands with copy-number aberrations can expand to form glandular clusters or mucosal patches and may contribute to field cancerization [11]. In longitudinal samples, however, progression is not explained by mutation burden alone. High-risk fields combine genomic alterations with lineage-state changes, altered transcription, inflammation, and microbial signals [12,14]. The somatic landscape of histologically normal gastric epithelium also shows that driver-bearing clones can exist without cancer [13]. Thus, the presence of a cancer-associated alteration identifies evolutionary potential, not an inevitable destination.

Longitudinal sampling strengthens temporal inference compared with a single resection, but serial biopsies usually resample an anatomical region rather than an identical gland. Failure to redetect a clone may therefore reflect sampling. Organoids allow functional perturbation, yet establishment and culture media select epithelial populations and remove much of the native immune, stromal, and microbial context. Neither design alone determines the prospective fate of every human gland [12,23].

The relationship between metaplasia and cancer is correspondingly heterogeneous. Some dysplasias are clonally continuous with adjacent IM, whereas spatially adjacent IM cannot always be assigned as the tumor’s direct clonal precursor [9,10,12]. Organoid studies further illustrate this heterogeneity: IM-derived cultures capture divergent epithelial-lineage trajectories and harbor distinct genetic and epigenetic features associated with early neoplastic progression [23]. Spatial transcriptomic, organoid, and epigenetic analyses of incomplete IM likewise reveal hybrid gastric–intestinal identities and substantial lineage plasticity [15]. Although these findings support the precursor potential of incomplete IM, molecular resemblance or spatial proximity to cancer does not, by itself, establish direct clonal ancestry; such a conclusion requires shared private alterations, phylogenetic reconstruction, or prospective lineage evidence.

Epigenetic field defects add a longer-lived layer of spatial memory. Aberrant methylation can persist after *H. pylori* eradication and is distributed beyond the visible tumor, creating a molecular field in which multiple independent neoplasms may arise [24,25,26,27,28]. Alterations affecting cell polarity, chromatin regulation, and tumor-suppressive pathways have also been detected in non-neoplastic or metaplastic mucosa [14,26,27,28,29]. These changes may alter the fitness landscape of glands before a morphologically recognizable lesion is present.

Multifocal gastric cancer illustrates why field-level reasoning matters. Whole-exome sequencing of synchronous gastric tumors has identified both shared and independent clonal origins: some lesions within the same stomach share multiple somatic alterations, whereas others show no detectable common mutations [30]. A common field exposure may therefore generate multiple altered gland populations, while individual tumors may either share a common ancestor or arise independently from distinct clones. The appropriate inference depends on assay resolution. Shared private mutations, concordant copy-number breakpoints, or a common phylogenetic trunk support clonal ancestry, whereas recurrent driver alterations, widespread epigenetic changes, or common exposure to *Helicobacter pylori* do not establish ancestry on their own.

Single-cell studies add epithelial state information to this clonal framework. Gastric premalignant lesions contain transitional, stem-like, and mixed-lineage epithelial populations that do not map neatly onto conventional categories such as normal glands, complete intestinal metaplasia, incomplete intestinal metaplasia, and cancer [16,31,32]. An integrated single-cell and spatial atlas of the human stomach further demonstrates that anatomical region, gland position, and epithelial–stromal interactions organize gastric homeostasis before neoplastic transformation [17]. In high-risk precursors, a spatially resolved 26-gene signature localizes to metaplastic glands and is expressed by aberrant mature and immature intestinal epithelial populations [18]. Experimental models further indicate that chronic inflammatory cues promote epithelial plasticity and can enhance the maturation and proliferation of metaplastic lineages [33,34,35].

### 2.2. Competing Explanations for Selective Progression

Selective progression has at least four, potentially interacting explanations. Clonal selection may favor repair and persistence without favoring malignancy; genomic instability increases the variation available for selection but does not define a sufficient transformation threshold [11,12,13,14]. Epigenetic alterations may stabilize abnormal differentiation or record prior inflammatory exposure, so methylation-based risk associations do not alone establish a causal mechanism [24,25,26,27,28,29]. Niche effects predict different outcomes among otherwise similar clones exposed to different local conditions. Conversely, an expanding clone may remodel its surroundings. Comparing clonally characterized glands across mapped environments before neoplasia, then perturbing the candidate exposure, would help distinguish these explanations. Apparent conflicts between studies should also be assessed against differences in lineage-marker resolution, sampling coverage, and selection of cancer-adjacent versus cancer-free tissue [9,10,12,30].

Together, these observations support an evolving-field model. Gland fission generates clonal patches; somatic and epigenetic alterations diversify gland populations; and spatially unequal inflammatory and niche signals may differentially favor their persistence and expansion. Any individual biopsy captures only a fragment of this process. The clinically relevant risk-bearing entity is therefore neither an isolated biopsy nor the stomach treated as a homogeneous organ, but a spatially structured population of glands with distinct clonal histories, epithelial states, and local microenvironments.

## 3. Cell-Intrinsic and Ecological Heterogeneity Within the Gastric Precancerous Field

### 3.1. Epithelial-State Heterogeneity and Cell-Intrinsic Alterations

Histology divides gastric precancer into atrophy, complete or incomplete IM, and dysplasia, but single-cell ribonucleic acid (RNA) data reveal a continuum of epithelial states within and between these categories. Early transcriptional maps identified stem-like, proliferative, absorptive, secretory, and cancer-associated populations across premalignant lesions and early gastric cancer [16,31]. The same biopsy can contain conventional gastric lineages, metaplastic cells with intestinal programs, and transitional cells that retain gastric markers while acquiring intestinal or wound-response genes [16,32]. This diversity provides a biological basis for differences between histologically similar biopsies, although cell-state differences alone do not establish different future cancer risks.

Metaplastic identity is not singular. Spasmolytic polypeptide-expressing metaplasia (SPEM) is studied in acute-injury and chronic-inflammation models that show shared metaplastic programs alongside context-specific epithelial responses [34,35]. In human IM, single-cell comparisons with esophageal metaplasia identify shared developmental programs, suggesting that injury-induced reprogramming reuses conserved epithelial modules [32]. Organoids derived from human IM retain divergent lineage trajectories and genetic backgrounds, providing an experimental system for comparing differentiation and features associated with neoplastic evolution [23].

Incomplete IM is particularly important because epidemiological risk and molecular plasticity converge. Spatial transcriptomics and organoid profiling show that incomplete IM can display a hybrid gastric, small-intestinal, and colonic identity across both differentiated and progenitor compartments [15]. Its association with cancer does not mean that every incomplete gland is a direct precursor. Rather, hybrid identity may indicate altered differentiation and responsiveness, although its stability and contribution to progression require direct testing [7,14,15,23].

A second high-risk feature is expansion of stem-like or proliferative compartments. Spatiotemporal profiling linked an intestinal stem-cell-dominant compartment to early malignancy [12]. Independent spatial work identified a 26-gene program in high-risk precursors at single-cell resolution [18], while experimental studies implicate sustained telomerase reverse transcriptase (TERT)-WNT/β-catenin activity in proliferative metaplastic states [36]. These signatures are promising because they move beyond the mere presence of IM, but their clinical value will depend on whether they predict future cancer after adjustment for extent, subtype, age, family history, and *H. pylori* status.

Validation differs between the leading studies. The 26-gene study included separate discovery and validation samples and orthogonal localization, but defined high-risk groups using advanced OLGIM stage; reproducing that contrast does not demonstrate prediction independent of OLGIM [18]. The IM-crypt study profiled eight specimens on four spatial captures, with direct IM-crypt–cancer continuity examined in one specimen. Its staining and external expression analyses support selected features, rather than prospective progression of the same gland [37].

Cell-state assignment also depends on where a cell is sampled. The normal human stomach contains strong regional and gland-axis programs [17]. A cell from the gland base and a cell near the surface may differ because of physiological position rather than malignant risk. Likewise, dissociation-based single-cell sequencing can obscure gland membership and remove extracellular context. Spatial confirmation is therefore necessary when a putative high-risk population is defined by proximity to dysplasia, a stem-cell niche, or a specific immune aggregate [15,16,17,18].

Reproducibility must be assessed at the patient level: many cells or spots from a few patients are not independent biological replicates. Spot mixing, cell segmentation, transcript assignment, targeted-panel coverage, fixation, anatomical sampling, and batch correction can change apparent states or neighborhoods [17,18,38,39]. Validation should prespecify the markers and analytical pipeline, hold out patients and centers, and confirm localization across platforms. Broad epithelial programs may reproduce while individual cluster labels do not.

The most defensible epithelial model is not a linear hierarchy of marker-defined cell types. It is a landscape containing multiple repair and differentiation trajectories. Somatic genotype constrains the routes available to a gland; epigenetic state alters their stability; and local morphogens, cytokines, metabolites, and microbes may influence which route persists [12,14,15,17,23,33,34,35,36,40,41,42]. Malignant risk is likely concentrated where a clone with relevant genomic capacity occupies a persistent, poorly differentiated, or highly proliferative state rather than in every gland that expresses an intestinal marker.

### 3.2. Immune, Stromal, and Vascular Microenvironments

A gland does not evolve in isolation. Its behavior depends on immune cells, fibroblasts, endothelial cells, extracellular matrix, and soluble signals arranged over tens to hundreds of micrometres. Single-cell atlases of gastric cancer demonstrate that epithelial lineage programs are embedded in distinct tumor microenvironments [19]. Importantly, profiling across precancer, localized cancer, and metastasis identifies stage-associated differences in immune and stromal states: IgA-positive plasma cells are abundant in premalignant tissue, whereas immunosuppressive myeloid and stromal populations become prominent later [20]. This is compatible with neighborhood succession, but stage-ordered samples from different patients do not directly observe that succession.

Early single-cell work documented extensive reprogramming of myeloid cells, T cells, endothelial cells, and fibroblasts in gastric cancer [43]. More recent analysis of IM and early cancer suggests that folate receptor beta (FOLR2)-positive macrophages decline during progression, although the direction and function of macrophage change vary by cohort and analytical definition [44]. These observations are best interpreted as changes in coordinated cell states, not as simple gains or losses of broad lineages. A macrophage marker, for example, does not specify whether the cell is tissue resident, inflammatory, phagocytic, angiogenic, or immunosuppressive.

The apparent contrast between loss of resident macrophage states in early lesions and enrichment of SPP1-positive or M2-like programs in established tumors need not be contradictory: stage, compartment, marker definitions, and sampling differ [20,44,45,46]. Marker loss may reflect displacement, while an M2-like expression score is not a direct assay of immune suppression. Neighborhood boundaries and patient-level effects require replication independently of tumor survival associations.

Fibroblasts are similarly heterogeneous. Across gastric carcinogenesis, fibroblast populations provide extracellular matrix, WNT and bone morphogenetic protein (BMP) modulators, chemokines, and growth factors that can support repair or constrain abnormal epithelium [17,20,40,45,46,47]. Spatially resolved gastric adenocarcinoma maps identify ecosystems in which fibroblast and myeloid programs co-localize with particular malignant states [48,49]. In advanced tumors, C-C motif chemokine ligand 2 (CCL2)-positive fibroblasts can participate in immunosuppressive crosstalk with signal transducer and activator of transcription 3 (STAT3)-activated macrophages, and other studies identify gremlin 1 (GREM1)-positive fibroblast–secreted phosphoprotein 1 (SPP1)-positive macrophage circuits [45,46]. These mechanisms should guide hypotheses in precancer, but they cannot be assumed to operate identically before invasion.

The term multicellular neighborhood is useful only when it remains operational. A neighborhood should specify a spatial radius or graph, the cell states included, their relative abundance, and the biological outcome being tested. Co-occurrence in a tissue section can generate hypotheses about ligand–receptor exchange, but inferred communication requires orthogonal evidence such as protein localization, receptor activation, perturbation in organoids or explants, or prospective association with progression. Studies that integrate spatial transcriptomics with histology and single-cell references provide a stronger starting point than dissociation data alone [17,18,22,45,48,50].

For gastric precancer, three candidate neighborhood classes deserve priority. The first is an inflammatory–metaplastic unit containing altered glands, macrophages, plasma cells, and cytokine-producing lymphoid cells [33,34,35,51,52,53]. The second is a stromal-remodeling unit in which fibroblast morphogens and matrix properties stabilize proliferative or hybrid epithelial states [17,22,40,45,46,47,48,50,54]. The third is a vascular-interface unit in which endothelial organization and perfusion may alter immune access and the conditions experienced by neighboring glands [19,20,22,43,48,55]. These boundaries may overlap; the analytical aim is not to force them into discrete boxes but to determine which combinations reproducibly predict clonal expansion or early invasion.

### 3.3. Helicobacter Pylori Gland Colonization and Atrophic Field Remodeling

*H. pylori* is often discussed as a luminal organism acting on the epithelial surface, but direct visualization has shown that it can enter gastric glands and interact with long-lived progenitor compartments. In antral glands, colonization activates and expands leucine-rich repeat-containing G-protein-coupled receptor 5 (LGR5)-positive stem cells, linking bacterial location to a specific epithelial response [56]. High-resolution mapping further revealed that colonization is organized into microniches rather than being evenly dispersed; gland architecture, local nutrients, and antimicrobial programs determine which niches remain permissive [57]. This gland-level topology provides a mechanistic bridge between persistent infection and the spatially focal emergence of altered epithelium.

The niche does not respond passively. Infection changes stem-cell turnover and induces epithelial and mesenchymal signals that remodel gland composition [56,58]. R-spondin signaling can drive secretory antimicrobial LGR5-positive cells, while stromal R-spondin controls stem-cell behavior and gland homeostasis [47,54]. In mouse and organoid systems, *H. pylori* can directly perturb human gastric epithelium, and chronic infection selects bacterial populations adapted to colonize metaplastic glands [59,60]. The result is reciprocal selection: bacteria adapt to altered glands as glands are reshaped by bacterial and inflammatory pressure.

Antral and corpus disease do not carry identical consequences. In the antrum, gland colonization can expand proliferative stem compartments; in the corpus, parietal-cell injury, acid loss, and altered morphogen gradients favour gland loss and metaplastic repair. Experimental work shows that gastric stem cells can promote inflammation and gland remodeling through an R-spondin 3 (RSPO3)-leucine-rich repeat-containing G-protein-coupled receptor 4 (LGR4) axis [40], whereas disruption of gland homeostasis changes the location and magnitude of bacterial colonization [40,57,59]. Human biopsy studies associate *H. pylori* infection with epithelial apoptosis that declines after eradication; this supports infection-associated injury without establishing toxin-specific causation [61].

The inflammatory compartment converts local injury into a field effect. Deletion of the Hedgehog effector Gli1 prevents *H. felis*-induced metaplasia and myeloid-subset expansion in mice, supporting a link between inflammatory signaling and epithelial remodeling in that model [52]. Single-cell analyses identify lineage-specific epithelial responses to chronic inflammation, including distinct chief-cell-derived and isthmal programs [34,35]. Group 2 innate lymphoid cells and macrophage-associated circuits coordinate damage responses, while interleukin-13 (IL-13) can promote the maturation and proliferation of metaplastic epithelium [33,53]. These observations place metaplasia within a multicellular repair program rather than treating it as an epithelial event alone.

Human disease introduces additional complexity. Autoimmune gastritis can generate oxyntic atrophy and metaplastic states without *H. pylori*, and cancer-related metaplastic cells have been detected across both etiologies [51]. Conversely, *H. pylori* may become sparse or undetectable after extensive atrophy, precisely when cancer risk is high. A negative test in an atrophic stomach therefore cannot be read as evidence that bacterial geography was never important; it may record the late stage of a field that the organism helped to create.

These infection and cytokine experiments establish effects on colonization, repair, and metaplastic growth in defined systems. They do not demonstrate that a particular colonized human gland will become cancerous, or that changes in epithelial turnover are equivalent to selection of a malignant clone.

A spatial model of *H. pylori*-associated carcinogenesis must therefore include at least three levels: occupation of individual gland niches, regional differences between antrum and corpus, and progressive remodeling of the whole-stomach field. Local bacterial presence is most informative early, when gland colonization and stem-cell responses can be measured directly. Later, the durable products of infection—gland loss, metaplastic expansion, methylation, altered immunity, and changed acid ecology—may dominate even after bacterial abundance declines [24,25,27,34,35,36,51,52].

### 3.4. Non-Helicobacter Pylori Microbial Geography

The gastric microbiome changes along the atrophy–metaplasia–cancer sequence, but abundance measured from a biopsy is not the same as spatial organization. Cancer-associated communities differ from those in non-neoplastic mucosa, with reduced dominance of *H. pylori* and enrichment of taxa often associated with the oral cavity [41]. After eradication, microbiota associated with inflammation, atrophy, and IM may persist, indicating that the remodeled gastric habitat continues to select a distinct community [62]. Oral and gastric samples from patients with IM also share selected taxa and functional pathways, supporting an oral–gastric connection without proving that oral organisms initiate metaplasia [63].

Several processes could generate microbial geography. Loss of parietal cells raises gastric pH, changes nitrate and bile-acid metabolism, and permits colonization by organisms poorly adapted to the healthy acidic stomach. Mucin composition and glycosylation differ between gastric, SPEM, complete IM, and incomplete IM glands; these changes alter attachment sites and nutrient availability. Gland depth, oxygen tension, luminal flow, and local immune effectors then create microniches within the same region [40,47,54,56,57,59,60]. Consequently, a biopsy-level community is an average of luminal contaminants, mucus-associated organisms, gland-associated bacteria, and organisms that may have crossed into the lamina propria.

Cross-sectional sequencing studies associate non-*H. pylori* dysbiosis with stages of gastric carcinogenesis, although individual taxa and diversity patterns vary across cohorts [41,64,65,66,67]. Longitudinal and functional evidence is more limited. Human gastric microbiota transplantation can reproduce premalignant lesions in germ-free mice, providing experimental support that community effects may extend beyond correlation [68]. Studies of paired oral and gastric sites show increasing concordance during carcinogenesis [69], while direct tissue imaging has detected bacterial invasion of the lamina propria in *H. pylori*-associated gastritis and precancer [70]. These results make spatial localization—not merely taxonomic detection—a central criterion for biological interpretation.

Reverse causation remains a central alternative: atrophy, acid loss, altered mucus, and occult cancer may permit secondary colonization while independently increasing cancer risk. Relative enrichment after loss of *H. pylori* dominance need not reflect increased absolute abundance. Community transplantation supports biological activity in a germ-free mouse model, but does not identify the responsible strain or metabolite or prove the same causal pathway in patients [41,62,68].

Microbial signatures may also carry clinical information. Gastric community profiles have been associated with metachronous recurrence after endoscopic resection [71], and combined methylation–microbiome models have been proposed to distinguish existing *H. pylori*-negative cancer from controls [72]. Such models are attractive because they sample field biology rather than the tumor alone. Yet performance must be tested across proton-pump inhibitor use, diet, geography, sequencing batch, oral health, prior antibiotics, and differing biopsy sites. Without these controls, a signature can encode site or cohort rather than disease mechanism.

These endpoints should remain separate: post-resection recurrence is not incident cancer in uncomplicated IM, and a casecontrol classifier does not establish prospective prediction [71,72]. Tissue imaging establishes location, not replication or viability. Extraction blanks, reagent controls, batch-balanced processing, and oral/luminal comparators are essential in low-biomass studies [73,74]. Culture can support viability where feasible, while a negative culture does not exclude living organisms; microbial RNA and viability-selective assays provide complementary evidence.

A rigorous spatial microbiome study of gastric precancer should therefore pair topographically mapped biopsies with mucus and luminal samples, quantify total biomass, include extraction and environmental controls, and localize candidate organisms by culture, targeted imaging, or spatial sequencing. It should also distinguish early gland-associated *H. pylori* from the broader communities that occupy an atrophic, hypochlorhydric field [41,56,57,59,63,68,70]. One testable model is succession: *H. pylori* helps construct the altered habitat, and a changing community may then maintain inflammation or metabolic stress after *H. pylori* itself becomes less abundant; secondary colonization remains a competing explanation.

Epstein–Barr virus (EBV) extends this spatial framework beyond bacteria. EBV-encoded small RNA (EBER) in situ hybridization distinguishes infected epithelial cells from infected cells in the inflammatory infiltrate; a positive bulk-tissue signal does not establish infection of a metaplastic gland [75]. Host and viral genomic analysis of 123 samples from 25 patients supported clonal continuity between EBV-positive high-grade dysplasia and carcinoma in selected lesions [76]. This strengthens lineage inference, but resection-based reconstruction does not determine when ordinary IM first becomes infected or establish a universal EBV-driven Correa sequence.

Experimental infection of immortalized, non-neoplastic gastric epithelial cells induced extensive de novo DNA methylation, supporting a direct epigenetic effect in that model without proving transformation of an intact human gland [77]. In established EBV-positive gastric cancers, programmed death-ligand 1 (PD-L1) expression in tumor and infiltrating immune cells and interferon-associated programs link viral status to the immune environment [78]. These tumor findings should not be assumed to describe a pre-existing precancerous niche. EBV is therefore considered a distinct viral route that may intersect with epithelial and ecological evolution; paired EBER, gland-lineage, methylation, and immune mapping is needed to resolve its timing and local effects.

### 3.5. Metabolic Gradients and Other Local Selective Pressures

Metabolic and physicochemical conditions form a further layer of local selection. Diffuse-type cancers contain microenvironments that vary with invasion depth [55], spatial metabolomics resolves tumor regions with distinct metabolite profiles [21], and integrated spatial multi-omics links cell-specific metabolic remodeling to local intercellular interactions [22]. Extrapolation to precancer must remain explicit because most available data come from established tumors. In an atrophic field, gland loss, reduced acid secretion, altered perfusion, hypoxia, immune metabolism, oxidative stress, bile-acid exposure, and microbial products could generate gradients that favor stress-tolerant epithelial states. The critical question is whether such gradients merely accompany damage or actively select clones capable of persistence and progression.

Microbial metabolism provides one bridge between community structure and epithelial selection. Deoxycholic acid can induce IM-associated signaling while altering bile-acid metabolism and the microbiota in experimental systems [42]. Integrated microbiome–metabolome studies identify stage-associated microbial functions and metabolites [79,80]. However, relative-abundance data are compositional, and metabolite detection alone does not identify its cellular source or effective tissue concentration. Absolute bacterial load, spatial localization, viability, and quantitative metabolite measurements are therefore needed before a taxon–metabolite pair can be assigned a causal role.

**Figure 1:**
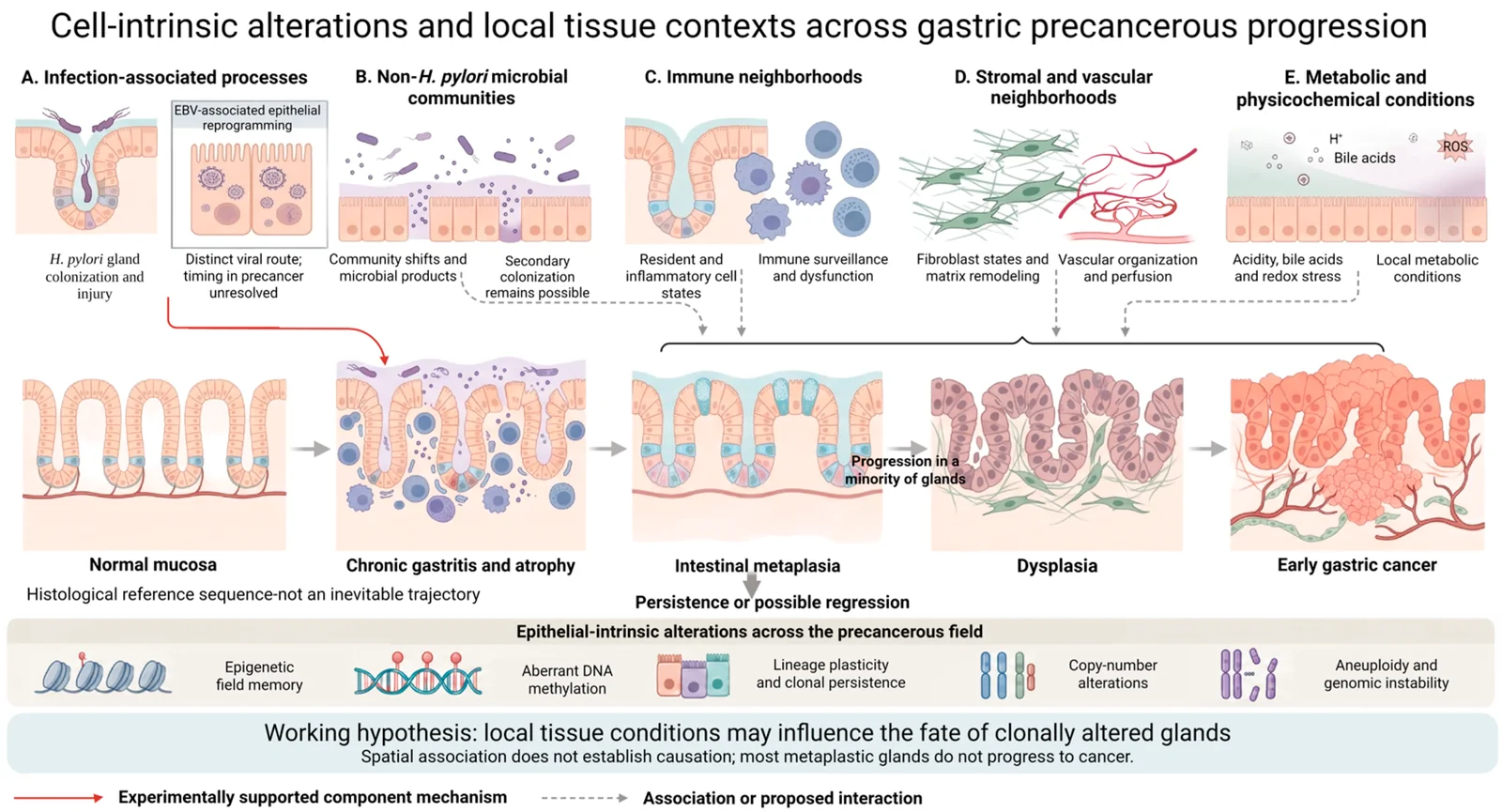
Cell-intrinsic alterations and local tissue contexts across gastric precancerous progression. The upper modules summarize infection-associated processes, non-*Helicobacter pylori* microbial communities, immune neighborhoods, stromal and vascular neighborhoods, and metabolic and physicochemical conditions. The middle row shows the classical histological reference sequence from normal mucosa through chronic gastritis and atrophy, intestinal metaplasia, dysplasia, and early gastric cancer; this sequence is not inevitable, and most metaplastic glands persist or regress rather than progress. The lower band summarizes epithelial-intrinsic alterations across the precancerous field. Gray solid arrows indicate the histological reference sequence, red solid arrows indicate experimentally supported component mechanisms in the cited settings, and gray dashed arrows indicate associations or proposed interactions. Epstein–Barr virus-associated epithelial reprogramming is shown as a distinct virus-associated route whose timing in precancer remains unresolved. The model is a working hypothesis: experimental support for an individual component does not establish the entire human progression pathway, and spatial association alone does not establish causation. Abbreviations: DNA, deoxyribonucleic acid; EBV, Epstein–Barr virus; *H. pylori*, *Helicobacter pylori*; ROS, reactive oxygen species. Created in BioRender.Kang, J. (2026) https://BioRender.com/iqcvou4 (accessed on 27 September 2026).

These noncellular influences should be treated as measurable exposures rather than as catch-all explanations. A candidate gradient should be quantified in mapped tissue, linked to a defined epithelial or neighborhood response, and tested through perturbation or longitudinal association. This standard is necessary to distinguish a causal selective pressure from a metabolic consequence of gland loss, inflammation, or incipient neoplasia (Figure 1).

## 4. From Spatial Precancerous Fields to Early Gastric Cancer

### 4.1. Spatial Continuity Versus Independent Tumor Emergence

The central evolutionary question is whether an early gastric cancer descends from a neighboring metaplastic or dysplastic clone or emerges independently within the same damaged stomach. Gland-level lineage studies provide direct evidence that some metaplastic and dysplastic glands share founder mutations and that dysplastic fields diversify into subclones [9,10]. Copy-number-aberrant IM can also expand over distance [11], and longitudinal sampling identifies persistent or evolving clones associated with later neoplasia [12]. In these cases, spatial continuity is biologically meaningful because it is supported by shared private alterations, not by adjacency alone.

Other observations support independent emergence. Histologically normal gastric glands already carry somatic mutations and selected clones [13]. Epigenetic field defects can affect broad mucosal regions [24,25,26,27,28,29], and synchronous tumors may be molecularly unrelated despite arising in the same stomach [30]. A common inflammatory and ecological field can therefore lower the threshold for multiple independent events. The field may supply shared selection pressures; it does not require a single ancestral precursor.

Field-level measurements do not uniformly explain local tumor emergence. The geospatial DNA-adduct study did not identify regional differences that accounted for tumor location [81]. Likewise, absence of shared mutations cannot establish independent origin when marker coverage or sampling is insufficient. Positive and negative lineage findings therefore require interpretation against assay sensitivity.

This distinction changes how cancer-adjacent IM should be described. A shared driver that is common in gastric epithelium is weak lineage evidence, whereas a constellation of shared private mutations, copy-number breakpoints, or mitochondrial variants is much stronger. Spatial transcriptomic similarity is still weaker because convergent cell states can arise in unrelated clones exposed to the same niche [12,15,16,17,18,23,32]. The most accurate conclusion is that gastric carcinogenesis permits both clonal continuity and independent tumor emergence, with their relative frequency still unresolved.

### 4.2. A Proposed Spatial Model of Malignant Transformation

A synthesis of the available data suggests six linked stages as a working model rather than a demonstrated temporal sequence. First, microbial colonization and chronic inflammation generate unequal injury across glands and gastric regions [34,35,40,52,53,56,57,59]. Second, gland loss, altered acid secretion, and stromal remodeling change the ecological niche [34,35,36,40,47,52,53]. Third, metaplastic clones expand by gland fission and accumulate genetic or epigenetic diversity [10,11,12,13,14,15,23,25,26,27]. Fourth, a subset enters high-risk epithelial states characterized by hybrid lineage identity, stem-like programs, proliferative persistence, or impaired differentiation [12,14,15,16,17,18,23,31,32,33,36]. Fifth, immune, stromal, vascular, metabolic, and microbial neighborhoods may select among those states [17,20,22,33,42,48,56,59,68,79]. Finally, only a minority of glands acquire sufficient cell-intrinsic capacity and local support to cross the threshold into dysplasia and invasion.

The model is deliberately non-deterministic. It allows a metaplastic clone to regress, persist, expand without transformation, or generate dysplasia. It also allows cancer to arise from a nearby but distinct gland lineage within the same field. In this view, the transition to early cancer is hypothesized to involve both clonal competence and local tissue support. The same alteration may behave differently across neighborhoods, a prediction requiring genotype-by-environment experiments.

Testing this model requires repeated, topographically registered sampling before cancer develops. Adjacent sections should be used for histology, gland-resolved deoxyribonucleic acid (DNA) sequencing, spatial ribonucleic acid (RNA) or protein mapping, and microbial localization. Organoids or explants derived from mapped glands can then test whether candidate stromal, immune, metabolic, or microbial factors alter persistence and invasion. Such designs would convert a descriptive spatial atlas into an evolutionary experiment and determine which features are causes, consequences, or durable records of earlier injury [12,15,17,18,21,22,23,33,45,48,51].

Although no single molecular alteration identifies a metaplastic gland that will inevitably progress, the evidence reviewed above suggests a set of features that may collectively indicate increased malignant potential (Table 1). These features occur at three interacting levels: intrinsic abnormalities within the gland, evidence of clonal persistence or expansion, and local ecological conditions that favor continued selection. Whether combining these features improves prediction beyond established staging remains to be demonstrated.

## 5. Clinical Translation: Spatial Risk Stratification and Surveillance

Clinical management already uses imperfect spatial surrogates. The extent and severity of IM, corpus involvement, and advanced OLGA or OLGIM stage predict cancer risk [3,4,5,6,7,82,83,84,85,86]. Systematic biopsy protocols therefore sample both antrum and corpus rather than relying on a lesion-directed specimen. This is not procedural formality: a limited set of biopsies can mis-classify a patchy field, and the same patient may move between risk categories when mapping is repeated more completely [8,87].

*H*. *pylori* eradication changes the future field but does not erase its past. Randomized and long-term studies show reductions in cancer or metachronous neoplasia in selected settings, yet patients with established atrophy, IM, or prior early cancer retain residual risk [88,89,90,91,92]. The apparent contradiction is resolved by timing. Removing the initiating organism may reduce ongoing injury, while expanded clones, methylation, and altered gland architecture persist. Surveillance decisions should therefore incorporate both current infection and accumulated field damage.

Progression estimates also depend on the endpoint. Incident cancer after a clean baseline examination, cancer detected soon after enrollment, and metachronous cancer after endoscopic resection are not interchangeable outcomes [82,83,85,88,89,90,91,92,93]. Early events may include lesions missed at baseline; metachronous tumors arise in a stomach already proven to support neoplasia. Biomarkers should be developed and validated for the clinical question they are intended to answer rather than pooling these outcomes into a single label of progression.

Endoscopic and histological staging can be strengthened by spatially informed imaging. Endoscopic grading of gastric intestinal metaplasia (EGGIM) and image-enhanced endoscopy estimate the visible extent of IM and may guide targeted biopsies [94,95,96,97]. Real-world studies support adherence to the updated Sydney mapping protocol, and recent work suggests that sampling along the lesser curvature can improve detection efficiency [87,98]. Nevertheless, optical classification and histology measure different aspects of the field. A practical strategy is to use endoscopy to map extent, histology to stage gland loss and IM subtype, and molecular assays only where they add reproducible discrimination.

Molecular risk models are now plausible. Genomic and epigenomic studies identify high-risk IM features [11,12,14], and spatial transcriptional signatures can localize precursor states associated with neoplasia [18]. Microbiome and combined methylation–microbiome models may add ecological information [71,72]. Before adoption, however, a new assay must improve prediction beyond age, family history, smoking, *H*. *pylori* status, IM extent and subtype, and OLGA/OLGIM stage. It must also tolerate the ordinary variability of biopsy site, fixation, endoscopist, and laboratory.

The reviewed evidence does not yet establish incremental clinical value for a gland-resolved risk map. A prospective study should enroll patients without neoplasia after a high-quality baseline examination, adjudicate incident high-grade dysplasia or cancer over a defined interval, and analyze early potentially missed lesions separately. Post-resection metachronous disease requires a separate validation setting. A locked model should be compared with prespecified clinical factors and OLGA/OLGIM, reporting calibration, discrimination, absolute risk, and decision-relevant net benefit in independent centers [3,8,18,25,71,72].

Surveillance intervals are necessarily a compromise between rare progression, imperfect sampling, procedure burden, and the opportunity to detect curable early cancer. Cohorts from low-incidence regions suggest that guideline-based surveillance can be safe for selected patients [99,100,101], while first-degree-relative screening studies highlight the importance of family history and population context [102]. Current European recommendations integrate extent, histological stage, incomplete IM, family history, and persistent infection [8]. A spatial framework does not replace these factors; it explains why they work and indicates where molecular refinement is most likely to help.

Feasibility and cost-effectiveness also remain unproven. A reduced assay should be tested for fixation sensitivity, failure rate, tissue consumption, turnaround time, and additional biopsy requirements. Decision-impact studies should measure changes in surveillance intervals, endoscopy burden, missed neoplasia, and stage at detection; economic evaluation should estimate incremental costs and quality-adjusted life-years. Analytical validity, predictive value, and improved clinical decisions are separate milestones.

**Figure 2:**
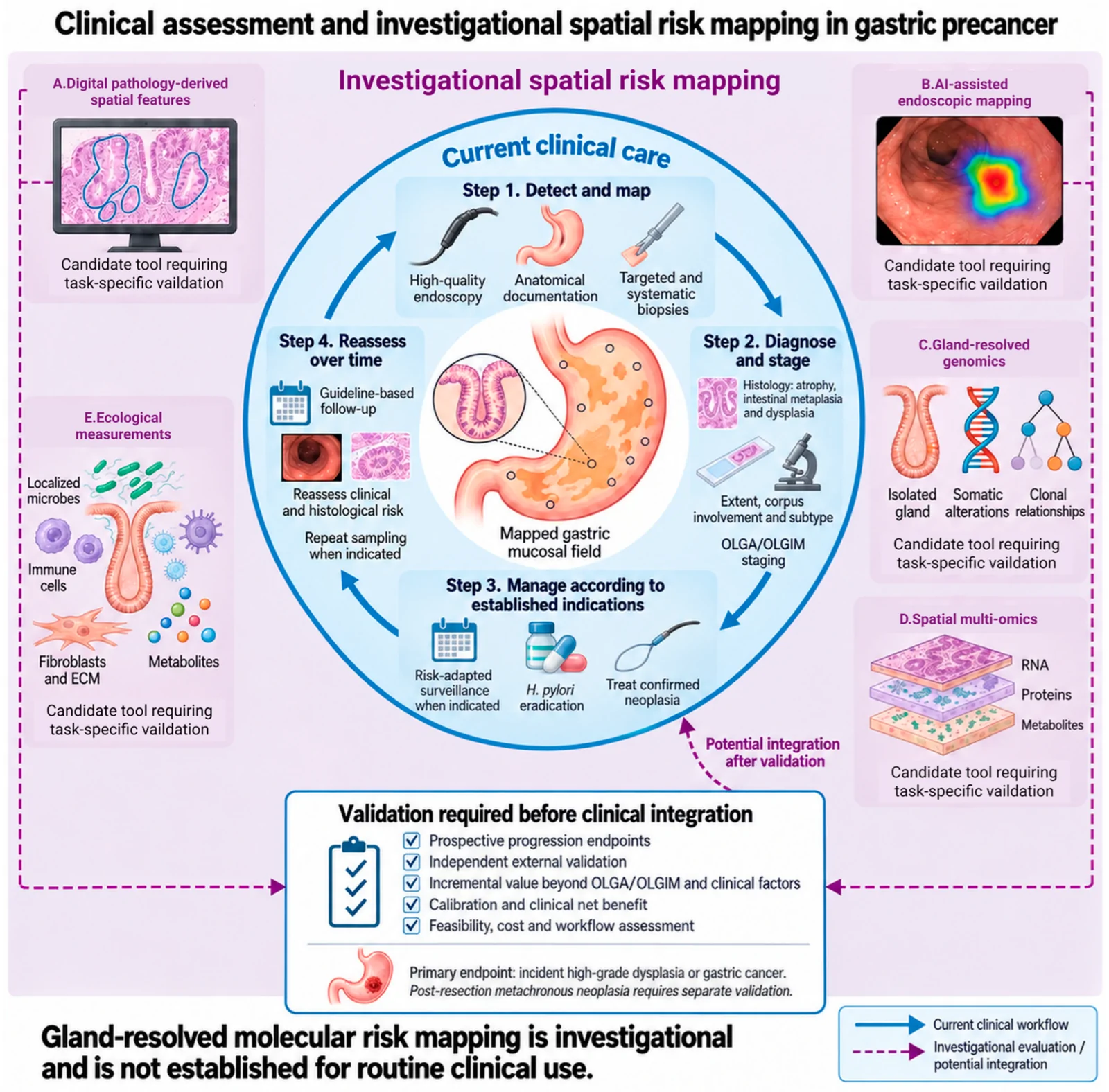
Clinical assessment and investigational spatial risk mapping in gastric precancer. The inner blue pathway summarizes current clinical care: high-quality endoscopic detection and anatomical mapping with targeted and systematic biopsy; histological diagnosis and staging based on atrophy, intestinal metaplasia, dysplasia, lesion extent, corpus involvement, subtype, and the Operative Link on Gastritis Assessment (OLGA) or Operative Link on Gastric Intestinal Metaplasia Assessment (OLGIM); management according to established indications; and guideline-based reassessment. The central stomach represents a mapped gastric mucosal field rather than a validated molecular risk map. The outer modules—digital pathology-derived spatial features, artificial intelligence-assisted endoscopic mapping, gland-resolved genomics, spatial multi-omics, and ecological measurements—are parallel investigational approaches requiring task-specific validation. Before clinical integration, studies should use prospective progression endpoints, independent external validation, comparison with OLGA/OLGIM and clinical factors, calibration and clinical net benefit, and feasibility, cost, and workflow assessment. Incident high-grade dysplasia or gastric cancer is the principal prospective endpoint; post-resection metachronous neoplasia requires separate validation. Solid blue arrows indicate the current clinical workflow, and purple dashed arrows indicate investigational evaluation or potential integration after validation. Gland-resolved molecular risk mapping is not established for routine clinical use. Abbreviations: AI, artificial intelligence; ECM, extracellular matrix; *H. pylori*, *Helicobacter pylori*; OLGA, Operative Link on Gastritis Assessment; OLGIM, Operative Link on Gastric Intestinal Metaplasia Assessment; RNA, ribonucleic acid. Created in BioRender. Kang, J. (2026) https://Biorender.com/un7q3hz (accessed on 27 Septmber 2026).

A near-term research goal is to evaluate a topographic risk map alongside established staging. Such a map would combine endoscopic extent, standardized antrum–incisura–corpus histology, gland-level molecular features, and a small set of validated epithelial or neighborhood markers. OLGA/OLGIM comparisons in cancer cohorts and regional gastritis indices show that anatomical distribution retains information beyond lesion presence [103,104]. Prospective trials should then test whether a spatially enriched model reduces missed high-risk fields or unnecessary surveillance without shifting error from one population to another. A practical implementation cycle is shown in Figure 2. Standard care includes endoscopic mapping, histopathological staging, and guideline-concordant management. Investigational approaches—such as gland-resolved molecular and ecological risk profiling—require prospective validation in well-designed trials.

## 6. Conclusions and Future Directions

The gastric precancerous field is best understood as a nested and evolving system. Somatic and epigenetic variation is organized among individual glands and clonal patches; epithelial cells occupy multiple repair and differentiation states; and immune, stromal, vascular, microbial, metabolic, and physicochemical factors may impose unequal local pressures. This framework accommodates both clonal continuity between metaplasia, dysplasia, and cancer and the independent emergence of tumors from different susceptible glands within the same injured stomach. Whole-stomach risk therefore arises from the spatial distribution and interaction of altered glands rather than from the most abnormal biopsy considered in isolation [10,11,12,13,15,17,20,56].

This interpretation remains constrained by the structure of the available evidence. Most human spatial studies are cross-sectional, frequently rely on gastrectomy specimens obtained after cancer has remodeled the tissue, and under-sample the topographic heterogeneity of the stomach. Spatial proximity cannot by itself establish common ancestry, temporal order, or causal signaling; transcriptional trajectories do not reconstruct a patient’s historical lineage; and platforms operating at spot, cellular, gland, or regional resolution measure different biological objects. Microbiome analyses face additional problems of low biomass, contamination, compositional abundance, and uncertain organism or metabolite localization, while cell-state and neighborhood nomenclature remains inconsistent across cohorts. Consequently, findings from established gastric cancer should be treated as hypotheses for precancer unless they are reproduced in appropriately staged tissue or supported by lineage, temporal, or functional evidence [9,12,38,39,73,74].

Progress will require longitudinal, topographically registered cohorts that sample chronic gastritis, atrophy, complete and incomplete intestinal metaplasia, dysplasia, and early intramucosal cancer with comparable methods. Adjacent sections should integrate standardized histology, gland-resolved genomic analysis, spatial RNA or protein mapping, and quantitative localization of microorganisms and metabolites, while preserving the hierarchy of cells within glands, glands within patches, and patches within anatomical fields. Because biopsy destroys the sampled gland, human follow-up tracks mapped fields and clone families rather than the identical gland. Private variants and structural breakpoints should anchor lineage reconstruction. Candidate interactions should then be perturbed in clonally characterized organoid, co-culture, explant, or lineage-traced animal systems, with appropriate controls, physiological exposures, and rescue where feasible. Persistence, expansion, dysplasia, or invasion should be measured alongside expression changes before a locked assay returns to independent human cohorts for validation. Such studies should incorporate sampling uncertainty, appropriate negative controls, absolute microbial load, relevant treatment exposures, and geographically diverse populations, and should compare any molecular model against established clinical staging rather than against normal tissue alone [3,8,18,23].

Clinically, the extent of intestinal metaplasia, corpus involvement, OLGA/OLGIM stage, and systematic endoscopic mapping already function as spatial surrogates of risk. The next advance is unlikely to come from replacing these measures with a single molecular marker; it will require demonstrating that gland-resolved clonal, epithelial-state, or neighborhood information improves prospective prediction and surveillance or prevention decisions. The proposed spatial framework therefore interprets gastric carcinogenesis from a uniform linear sequence into a non-deterministic process in which chronic injury creates a permissive field, metaplastic clones expand and diversify, and only a minority of glands encounter the molecular competence and local ecology required for malignant transformation [5,7,8,18].

## Figures and Tables

**Table 1:** Candidate gland-level and neighborhood indicators of increased malignant potential in gastric precancer.

Candidate Indicator and Unit	Biological or Clinical Interpretation	Evidence Status	Refs.
**Part A. Established clinicopathological indicators used in diagnosis or risk stratification**			
Histological dysplasia Unit: gland/lesion	Architectural complexity, loss of polarity, gland fusion, and cytological atypia indicate established neoplastic transformation rather than susceptibility alone.	Clinically established. Grade and expert pathological confirmation directly affect management; this is diagnostic evidence rather than an experimental biomarker.	[4,8,82]
Extent and topographic distribution of atrophy or IM Unit: patient/mucosal field	Extensive or corpus-involving disease and advanced OLGA/OLGIM stages indicate a larger and more severely remodeled field in which high-risk glands may arise.	Clinically established at the patient level. It stratifies surveillance risk but does not identify the fate of an individual gland.	[3,5,8,83]
Incomplete IM subtype Unit: lesion/patient	Incomplete IM is associated with higher patient-level cancer risk. This clinicopathological association should be distinguished from exploratory hybrid molecular identities.	Clinically supported as a histological risk factor. Independent prediction beyond extent and stage varies across populations; hybrid molecular identity remains exploratory.	[7]
**Part B. Investigational gland-level and ecological features**			
Gland clonality, persistence and spatial expansion Unit: gland/patch	Related gland patches, gland fission, copy-number-aberrant expansion, or persistence across serial samples increase the population and time available for further selection.	Directly observed in human gland-lineage and genomic studies, but not validated as a prospective predictor of an individual gland’s fate.	[9,10,11,12]
Genomic alterations and driver evolution Unit: gland/patch	Increasing copy-number alteration burden, aneuploidy, and selected driver events may mark movement toward neoplastic competence; driver-bearing clones can also persist without cancer.	Human molecular and longitudinal association. No alteration-specific transformation threshold or surveillance-biopsy positive predictive value is established.	[11,12,13,14]
Epigenetic field burden Unit: mucosal field, occasionally gland	Persistent aberrant DNA methylation may record cumulative inflammatory injury and stabilize abnormal lineage programs, but can reflect field exposure rather than the fate of a sampled gland.	Selected markers have prospective patient-level associations, particularly in post-resection settings; gland-specific and general-population predictive performance remains insufficiently defined.	[14,15,24,25]
Hybrid identity and high-risk epithelial programs Unit: epithelial cell/gland	Hybrid, stem/progenitor-like, proliferative, OLFM4-positive, and high-risk transcriptional programs are enriched in advanced precursors and may identify abnormal metaplastic glands.	Expression and localization are supported for selected programs. The 26-gene study used OLGIM-defined risk groups; independent prospective prediction remains unestablished.	[14,15,16,18,37]
Persistent *H*. *pylori* gland colonization and epithelial responses Unit: gland/mucosal field	Deep gland colonization, virulence-factor delivery, and R-spondin–LGR-dependent responses can sustain focal inflammation, stem-cell activation, and remodeling.	Localization and component mechanisms are supported by human observations and experimental models; not validated as a gland-level prognostic marker.	[40,56,57,59]
Non-*H*. *pylori* community changes Unit: field/local neighborhood	Oral-derived or other communities may accompany atrophy and contribute microbial products or inflammatory signals.	Mainly human association, with functional support from germ-free transplantation. Reverse causation, contamination, viability, and responsible taxa remain unresolved.	[41,68,79]
Metabolic and physicochemical exposures Unit: field/local neighborhood	Reactive oxygen species, bile acids such as deoxycholic acid, hypoxia, acidity, and microbial metabolites may injure tissue or select stress-tolerant epithelial states.	Experimental effects exist for selected exposures; broader spatial gradients remain associative or hypothetical in human precancer.	[42,79]
Immune–stromal neighborhoods and ecological selection Unit: cellular neighborhood	Loss of resident macrophage states, SPP1-positive or M2-like programs, activated fibroblasts, remodeled ECM, and altered vascular interfaces may influence epithelial persistence.	Exploratory in human precancer. Several named circuits are demonstrated mainly in established cancer; selection of malignant human precancer clones remains a hypothesis.	[20,44,45,46]

Abbreviations: DNA, deoxyribonucleic acid; ECM, extracellular matrix; *H. pylori*, *Helicobacter pylori*; IM, intestinal metaplasia; LGR, leucine-rich repeat-containing G-protein-coupled receptor; OLFM4, olfactomedin 4; OLGA, Operative Link on Gastritis Assessment; OLGIM, Operative Link on Gastric Intestinal Metaplasia Assessment; SPP1, secreted phosphoprotein 1. Clinical maturity is specific to the stated endpoint and population; expression replication, tissue localization, experimental function, and prospective outcome prediction are distinct forms of validation. No combined gland-level cutoff or ecological score is validated by this review.

## Data Availability

No new data were created or analyzed in this study. Data sharing is not applicable to this article.
